# Enhanced Anti-Inflammatory Effects of Silibinin and Capsaicin Combination in Lipopolysaccharide-Induced RAW264.7 Cells by Inhibiting NF-κB and MAPK Activation

**DOI:** 10.3389/fchem.2022.934541

**Published:** 2022-06-30

**Authors:** Yingying Zheng, Jie Chen, Xiaozheng Wu, Xin Zhang, Chunmei Hu, Yu Kang, Jing Lin, Jiamin Li, Yuechang Huang, Xingmin Zhang, Chen Li

**Affiliations:** School of Biotechnology and Health Sciences, Wuyi University, Jiangmen, China

**Keywords:** anti-inflammatory, silibinin, capsaicin, NF-κB, MAPK

## Abstract

Silibinin and capsaicin both are natural product molecules with diverse biological activities. In this article, we investigated the anti-inflammatory effects of silibinin combined with capsaicin in lipopolysaccharide (LPS)-induced RAW264.7 cells. The results showed that silibinin combined with capsaicin strongly inhibited LPS-induced nitric oxide (NO), tumor necrosis factor-α (TNF-α), Interleukin-6 (IL-6), and COX-2. Moreover, silibinin combined with capsaicin potently inhibited nuclear factor-κB (NF-κB) and mitogen-activated protein kinase (MAPK) signaling pathways. The results of the present study indicate that silibinin combined with capsaicin effectively inhibits inflammation.

## Introduction

Inflammation is confirmed to be related to several progressive diseases (Irmscher et al., 2019; Bai et al., 2021), such as metabolic disorders (Bai et al., 2021), cancer, obesity (Kase et al., 2020), and cardiovascular disease. Thus, to eliminate or prevent inflammation is very necessary to maintain body health. Inflammatory cells are activated in the inflammatory process and release high levels of pro-inflammatory cytokine (Luo et al., 2019; Huang et al., 2022), including interleukin (IL)-1β, IL-6, nitric oxide (NO), and tumor necrosis factor-α (TNF-α), causing tissue damage and involve in the development of various diseases (Zhao et al., 2021). NO is an important pro-inflammatory mediator that is regulated by cyclooxygenase-2 (COX-2) and inducible nitric oxide synthase (iNOS) (Akanda et al., 2018). The secretion of pro-inflammatory mediators, such as COX-2, iNOS, IL-1β, IL-6, and TNF-α, is mediated by transcription factor nuclear factor-κB (NF-κB) (Wang et al., 2022). The NF-κB protein is composed of two subunits (P50 and P65) and bound to the inhibitor of NF-κB (IκB) to an inactive state (Yu et al., 2020). When cells are stimulated by endogenous and exogenous, IκB causes phosphorylation and degradation, resulting in the release of NF-κB and subsequently phosphorylation of p65 (Cai et al., 2021). Then, NF-κB is translocated to the nucleus from the cytoplasm and regulate the target gene (Chen et al., 2019). Moreover, mitogen-activated protein kinase (MAPK) signaling pathways are another important protein to regulate the inflammation process (Lee H. -J. et al., 2018; Zhao et al., 2021). MAPK pathway, composed of p38, ERK, and JNK, can also be activated by various stimuli. MAPK pathway is closely related to the NF-κB activation (Zhao et al., 2021). Thus, inhibiting NF-κB and MAPK pathways is the effective strategy to suppress the inflammatory response (Zhou et al., 2018; Moon et al., 2019).

Silibinin, the major active constituent of *Silybum marianum*, is one kind of polyphenolic flavonoid that has been used as medicine for a long time and is well known for its hepatoprotective and anti-carcinogenic effects (Hussain et al., 2020; Fallah et al., 2021). Moreover, silibinin, has been reported to have effective anti-inflammatory effects (Chen et al., 2020). Silibinin could down-regulate pro-inflammatory mediators in various inflammation-related disease models by the suppression of signaling pathway activations, such as NF-κB, MAPK, and STAT-3 (Rengasamy et al., 2019; Villegas-Aguilar et al., 2020). On the other hand, capsaicin is the active ingredient in chili peppers (Li et al., 2020) that has been used in lots of pharmacological researches to reveal its various physiological processes, such as cardiovascular (Wang et al., 2021), respiratory (Hernández-Pérez et al., 2020), and inflammation (Wang et al., 2021). Now, capsaicin has been verified to attenuate inflammation in some inflammation models (Hernández-Pérez et al., 2020).

Up to now, combination therapy has been considered as an effective strategy to attenuate inflammation (Bruni et al., 2018). The combination of different anti-inflammation agent results in the enhancement of pharmacological activity by acting on multi-target t(Hong et al., 2021). A combination of silibinin with brostallicin could increase the anti-apoptotic protein Bcl-2 and decrease in caspase 3 activity (Pook et al., 2006). Silibinin in combination with Pu-erh tea extract could relieve non-alcoholic fatty liver disease (Hu et al., 2017). In this study, the enhanced anti-inflammatory effects of the silibinin and capsaicin combination were investigated in LPS-induced RAW264.7 cells.

## Experimental

### Materials

Silibinin (purity ≥98% by HPLC) and capsaicin (purity ≥98% by HPLC) were purchased from Shanghai Yuanye Biological Technology Co., Ltd. (Shanghai, China). Fetal bovine serum (FBS) and Dulbecco’s Modified Eagle’s Medium (DMEM) were purchased from Gibco (Grand Island, NY, United States). Lipopolysaccharide (LPS, from *Escherichia coli* O111:B4) and 3-(4,5-dimethythiazol-2-yl)-2,5-diphenyl-tetrazoliumbromide (MTT) dye were obtained from Sigma-Aldrich (St. Louis, MO, United States). Antibodies against COX-2 (12282S), p-JNK (9251S), p-ERK (9101S), ERK (9102S), p-p38 (9211S), p38 (9212S), and p65 (8242S) were purchased from Cell Signaling Technology (Danvers, MA, United States). The IL-6 (mouse) ELISA Kit and murine TNF-α Pre-Coated ELISA kit were purchased from Cayman Chemical Co., Ltd. (Ann Arbor, MI, United States). The Griess reagent (modified) was obtained from Sigma-Aldrich. NE-PER Nuclear Extraction Kit was obtained from Thermo Fisher Scientific Inc. (Waltham, MA, United States).

### Cell Culture

RAW264.7 cells were obtained from the American Type Culture Collection (ATCC, Rockville, MD, United States). The cells were cultured in DMEM containing with 10% FBS and 1% P/S in an incubator maintained at 37°C under humidified atmospheric conditions consisting of 5% CO_2_.

### Cell Viability Assay

RAW264.7 cells (5,000 cells/well) were seeded in 96-well plates and cultivated for 24 h. After silibinin and capsaicin with different concentrations were added for 2 h, the cells were treated with or without LPS (1 μg/ml) for 24 h. To discard the medium, MTT reagent was added, and incubated for 3 h. Formazan crystals were dissolved in 100 μL of DMSO, and then absorbance at 490 nm was measured.

### NO Assay

After being plated into 96-well plates and incubated for 24 h, the cells were treated with silibinin and/or capsaicin for 2 h and subsequently co-treated with LPS (1 μg/ml) for 24 h. The medium was mixed with the Griess reagent (1: 1) and placed in the dark for 15 min. Then, the absorbance was measured at 540 nm.

### Synergistic Effect Analysis

The synergistic effect between silibinin and capsaicin was analyzed based on NO assay data by the CompuSyn software 2.0. The NO assay data of silibinin and capsaicin alone or the combination were entered into the CompuSyn software, then combination index (CI) values could be produced in the default mode. The CI values revealed the additive, synergism, or antagonism of the combinations of the two drugs at different doses.

### ELISA Assay

After being plated into 24-well plates for 24 h and the cells were treated with silibinin and/or capsaicin for 2 h and subsequently induced with LPS (1 μg/ml) for 24 h. Then, the supernatant was detected for TNF-α and IL-6 levels according to the manufacturer’s instructions.

### Western Blot Analysis

After RAW264.7 cells (100,000 cells/dish) were cultured in a 100 mm dish for 24 h, cells were treated with silibinin and/or capsaicin for 2 h, and then treated with LPS (1 μg/ml). Then cells were collected, lysed with lysis buffer for 10 min at 4°C, and centrifuged for 10 min at 12,000 g to obtain supernatant. After determining the protein concentrations, supernatant was boiled with loading buffer for 10 min. Then the sample was separated by SDSPAGE (8%) and transfer to the nitrocellulose membrane, followed by the block for 1 h in non-fat milk (5%), incubated with the primary antibodies (COX-2, NF-κB p65, p-p38, p38, p-ERK, ERK, p-JNK, β-actin, and Lamin B) overnight at 4°C, and incubated with the secondary antibody for 1 h. Then, the sample was visualized by enhanced chemiluminescence (ECL) detection kits.

### Statistical Analysis

Data were expressed as the mean ± SEM. We used the SPSS software to perform the statistical analysis. Data were analyzed using the one-way ANOVA. The results were considered statistically significant when *p* < 0.05.

## Results and Discussion

### Cytotoxicity Assay of Silibinin and Capsaicin

**FIGURE 1:**
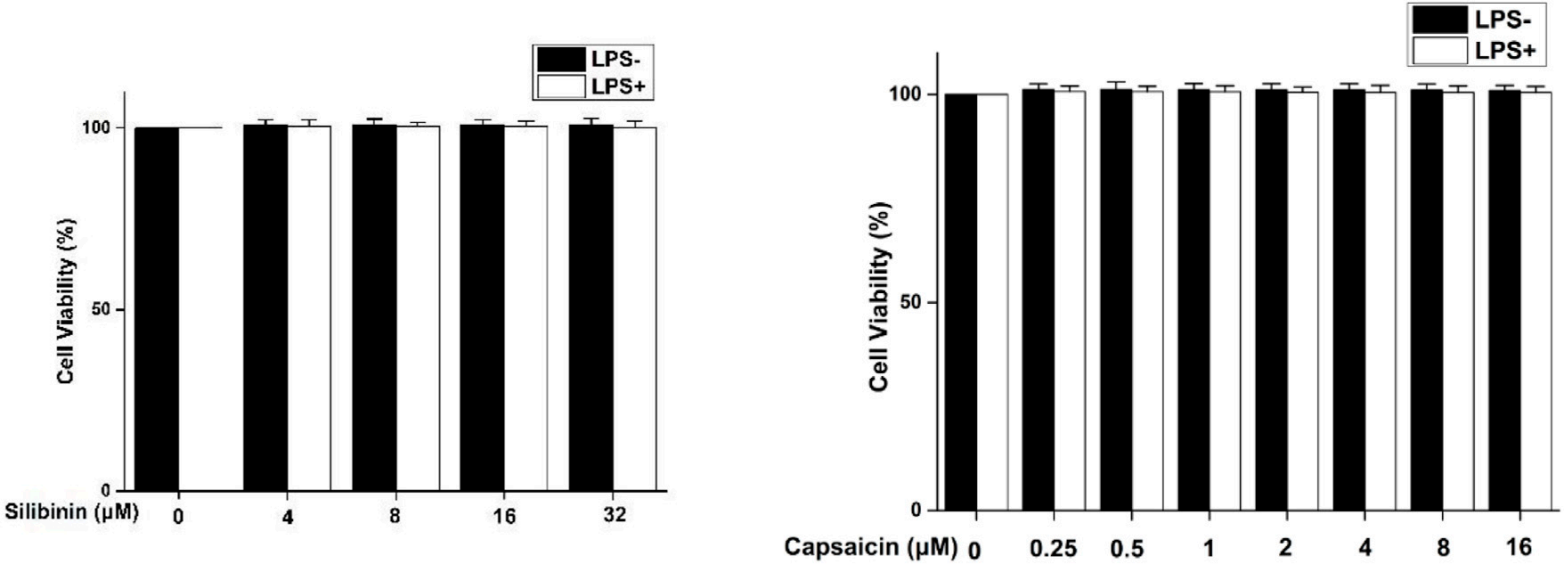
Cytotoxicity of silibinin and capsaicin on RAW264.7 cells. RAW264.7 cells were seeded in 96-well plates and cultivated for 24 h. After silibinin and capsaicin with different concentrations were added for 2 h, the cells were treated with or without LPS (1 μg/ml) for 24 h. To discard the medium, the MTT reagent was added , and incubated for 3 h. Formazan crystals were dissolved in 100 μL of DMSO, and then absorbance at 490 nm was measured.

The cytotoxicity assay of silibinin and capsaicin was firstly evaluated using the MTT assay and the results are shown in Figure 1. It could be seen that silibinin (4–32 μM) and capsaicin (0.25–16 μM) showed no cytotoxicity to RAW264.7 cells both with or without the stimulation of LPS.

### Effects of Silibinin and Capsaicin on LPS-Induced NO

**FIGURE 2:**
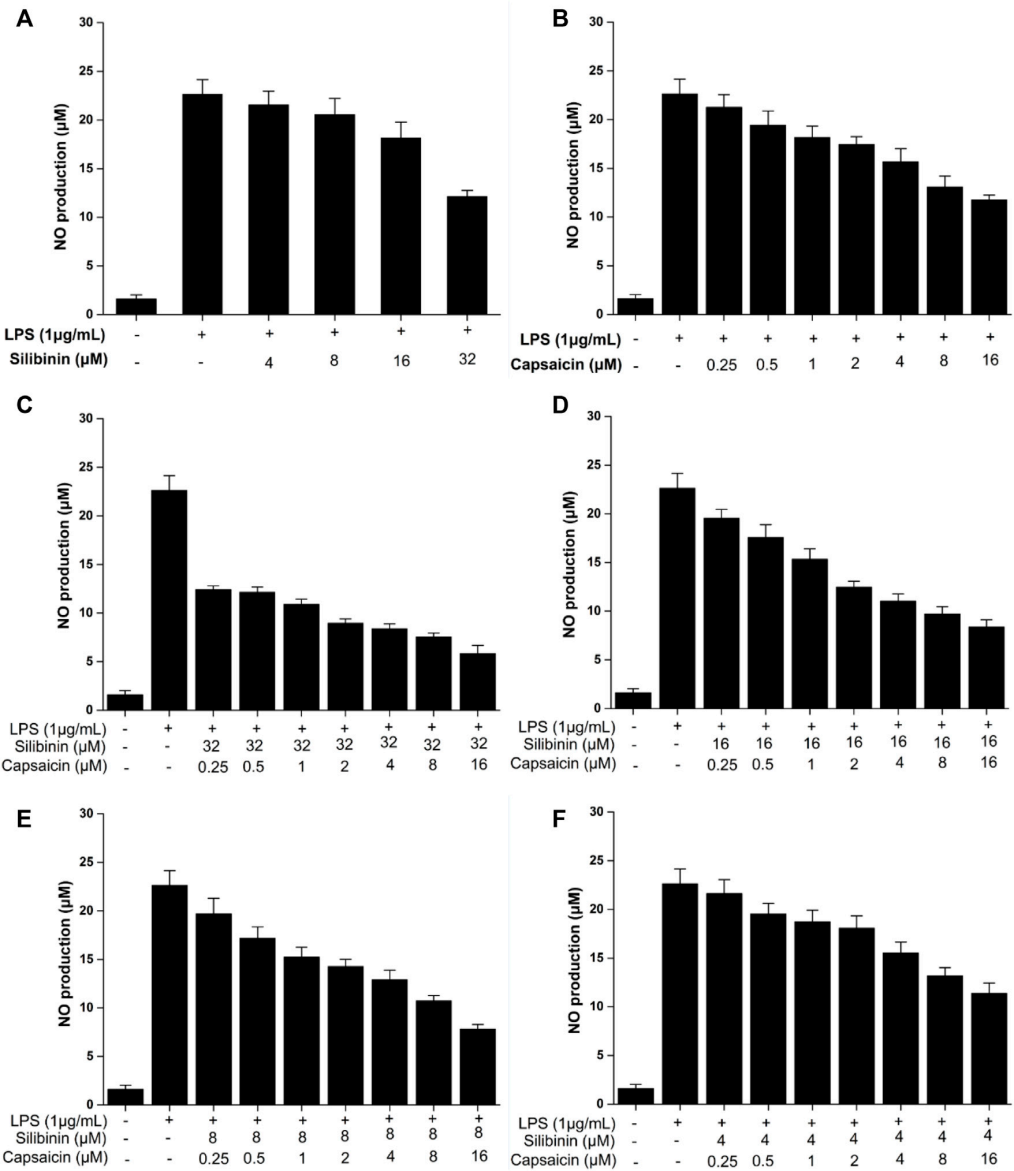
Inhibitory effect of silibinin (4–32 μM) on the LPS-induced NO **(A)**. Inhibitory effect of capsaicin (0.25–16 μM) on the LPS-induced NO **(B)**. Inhibitory effect of silibinin and capsaicin combination at different concentration ratios on the LPS-induced NO **(C–F)**. After being plated into 96-well plates and incubated for 24 h, the cells were treated with silibinin and/or capsaicin for 2 h and subsequently co-treated with LPS (1 μg/ml) for 24 h. The medium wasmixed with the Griess reagent (1:1) and placed in the dark for 15 min. Then the absorbance was measured at 540 nm.

NO, secreted at inflammatory sites, is the important cellular mediato (Sugimoto et al., 2019). The effect of silibinin and capsaicin on the LPS-induced NO production was assayed. The results (Figure 2) showed that silibinin (4–32 μM) and capsaicin (0.25–16 μM) could effectively inhibit NO production induced by LPS (Figures 2A,B). The silibinin and capsaicin combination presented stronger inhibitory than silibinin or capsaicin alone (Figures 2C–F), suggesting that there was a synergistic effect between silibinin and capsaicin.

### Synergistic Effect Analysis

**FIGURE 3:**
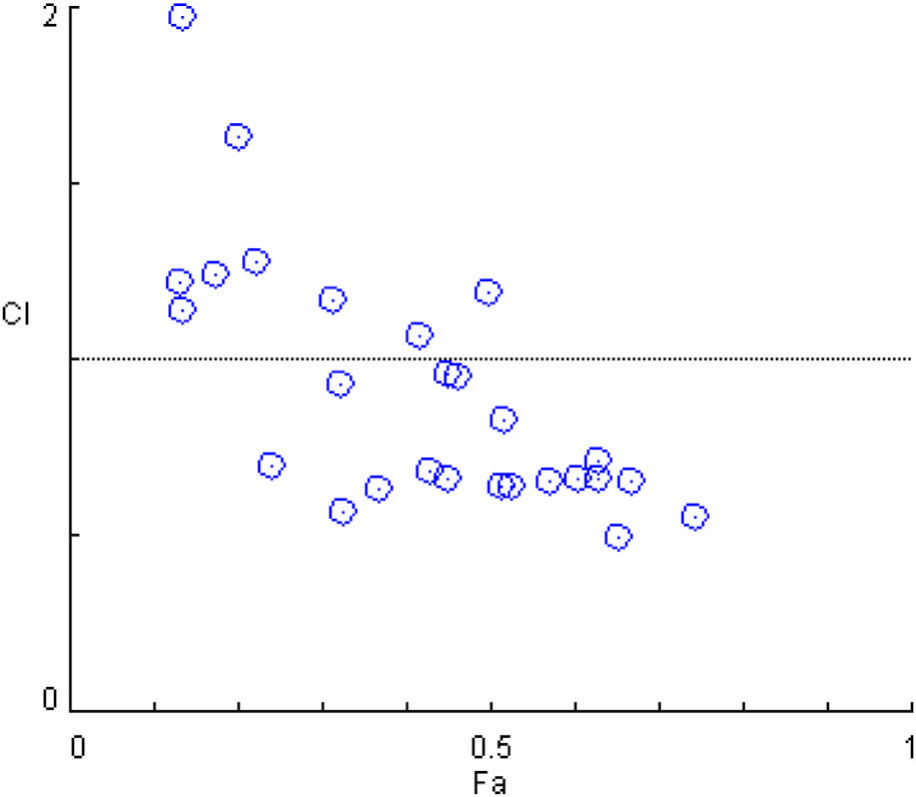
Combination index analysis of the silibinin and capsaicin combination.

The synergistic effect of silibinin and capsaicin was analyzed based on the results of NO assay data. The CI value is a quantitative parameter on the degree of drug interaction, including synergism, additive effect, and antagonism. Cl < 1 indicated that there was synergistic effect, and the lower CI means the higher synergistic effect. Figure 3 presented the CI values calculated by the CompuSyn software 2.0. The Silibinin and capsaicin combination had CI values ranging from 0.50 to 3.80. Among them, silibinin (8 μM) and capsaicin (16 μM) combination had the lowest CI value of 0.50, meaning that silibinin (8 μM) and capsaicin (16 μM) combination had the best synergistic effect. Then, the mechanistic studies of The silibinin and capsaicin combination were investigated using the optimal dose ratio.

### Effect of Silibinin and Capsaicin on TNF-α and IL-6

**FIGURE 4:**
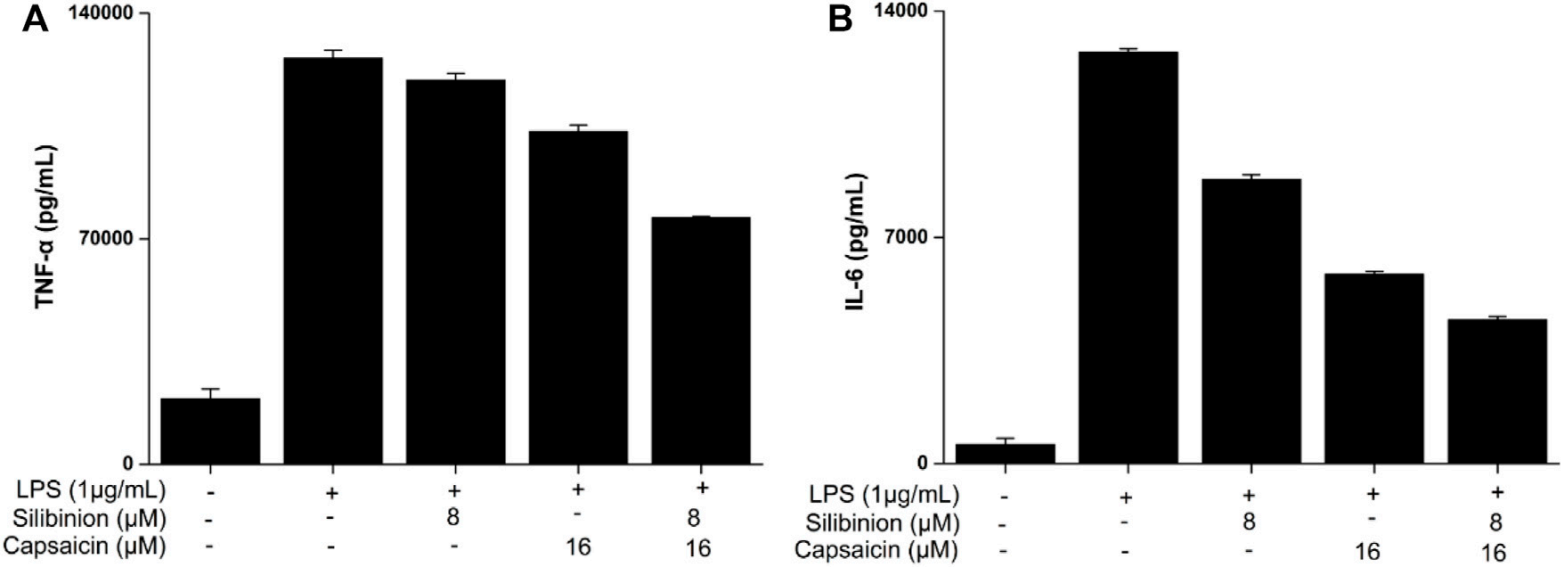
Effect of silibinin and capsaicin on LPS-induced TNF-α **(A)** and IL-6 **(B)**. After being plated into 24-well plates for 24 h and the cells were treated with silibinin and/or capsaicin for 2 h and subsequently induced with LPS (1 μg/ml) for 24 h. Then, the supernatant was detected for TNF-α and IL-6 levels according to the manufacturer’s instructions.

The secretion of pro-inflammatory cytokines, such as TNF-α and IL-6, is important to relieve exogenous stimuli and repair the injured tissues (Yang et al., 2020). So, the effect of silibinin and capsaicin on TNF-α and IL-6 was detected using ELISA assay and the results are shown in Figure 4. Treatment with silibinin and capsaicin alone could effectively inhibit the LPS-induced TNF-α and IL-6. While, the silibinin and capsaicin combination showed a stronger inhibitory effect on TNF-α and IL-6 than silibinin and capsaicin alone.

### Effect of Silibinin and Capsaicin on LPS-Induced COX-2

**FIGURE 5:**
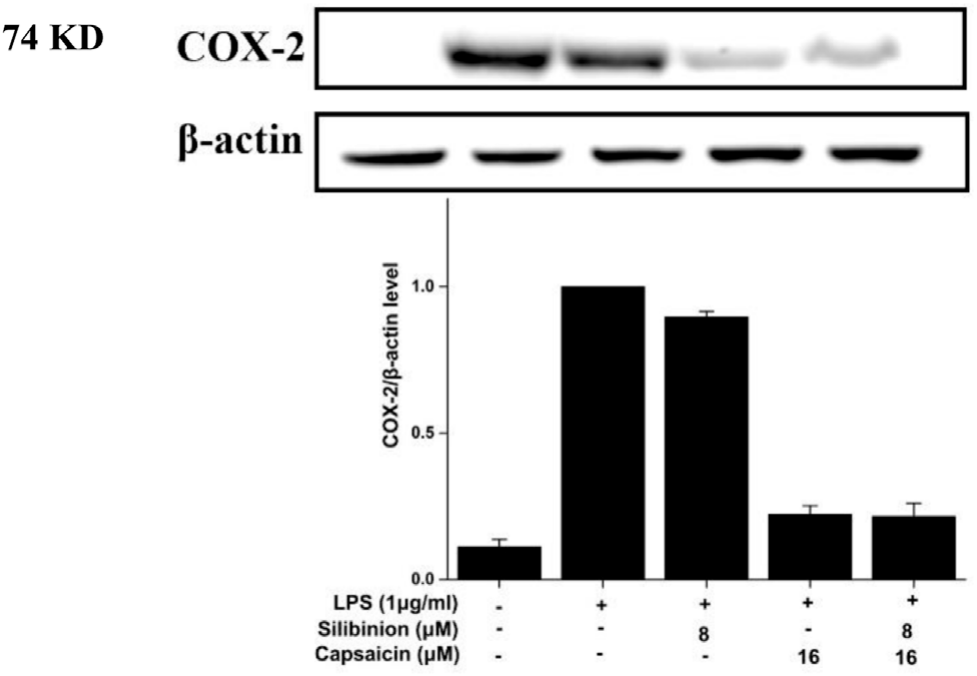
Effect of silibinin and capsaicin on the LPS-induced COX-2. After separated by SDSPAGE (8%), transfer to the nitrocellulose membrane, and block for 1 h in non-fat milk (5%), the sample was incubated with the primary antibodies COX-2 overnight at 4°C, and subsequently incubation with the secondary antibody for 1 h.

COX-2 has been reported to play an important role in the inflammatory process (Wang et al., 2019). Based on the significant inhibition of NO by silibinin in combination with capsaicin or alone, their effect on COX-2 was assayed. As shown in Figure 5, LPS treatment obviously induced and increased COX-2 expression. Treatment with silibinin and capsaicin alone could inhibit the COX-2 expression with a reduction of 12.5 and 76.9%, respectively. The Silibinin and capsaicin combination obviously suppressed the COX-2 expression with a reduction of 76.9%. The results showed that capsaicin (16 μM) had stronger effect than silibinin (8 μM), while the combination did present higher inhibition that capsaicin. That is to say that the combination of silibinin and capsaicin had little enhancement on the reduction of COX-2.

### Effect of Silibinin and Capsaicin on the LPS-Induced NF-κB Pathway

**FIGURE 6:**
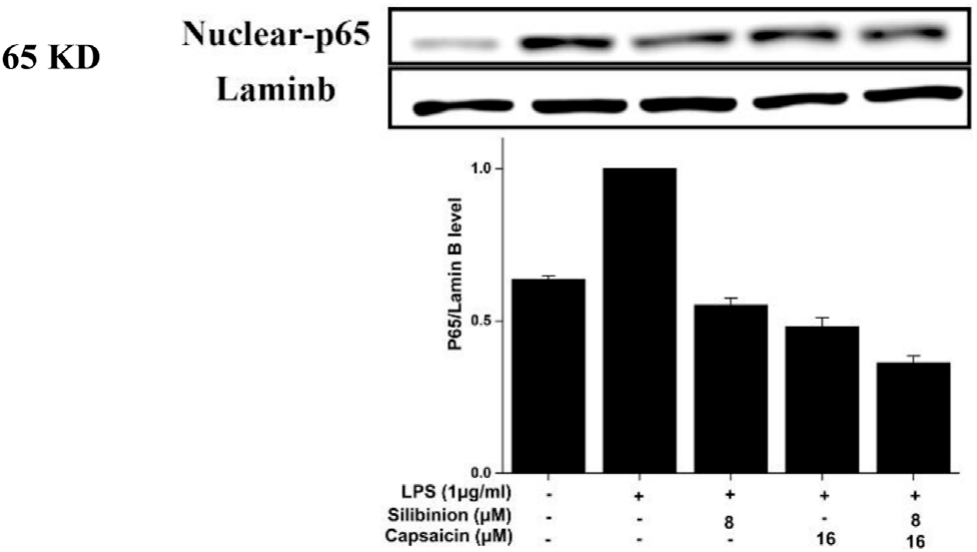
Effect of silibinin and capsaicin on the LPS-induced NF-κB pathway. After separated by SDSPAGE (8%), transfer to the nitrocellulose membrane, and block for 1 h in non-fat milk (5%), the sample incubated with the primary antibodies p65 overnight at 4°C, and subsequently incubation with the secondary antibody for 1 h.

NF-κB, an essential transcription factor, was involved in proinflammatory responses. When stimulated, NF-κB (p65) was released from its bound complex and translocated into the nucleus from the cytosol, then regulating pro-inflammatory genes production (Lee S. -B. et al., 2018). As observed from Figure 6, silibinin and capsaicin pre-treatment alone could down-regulate the activation of LPS-induced NF-κB (p65) by 44.8 and 51.7%, respectively. Simultaneously, the silibinin and capsaicin combination obviously down-regulated the NF-κB activation by 62.1%, which was higher than that of silibinin and capsaicin alone, revealing the synergistic effects of the two compounds. Chen et al.‘s research revealed that silibinin could enhance the inhibitory effect of thymol on LPS-induced NF-κB (p65) activation (Chen et al., 2020). Our results revealed that silibinin also improved the inhibitory effect of capsaicin on LPS-induced NF-κB (p65) activation.

### Effect of Silibinin and Capsaicin on LPS-Induced MAPK Pathway

**FIGURE 7:**
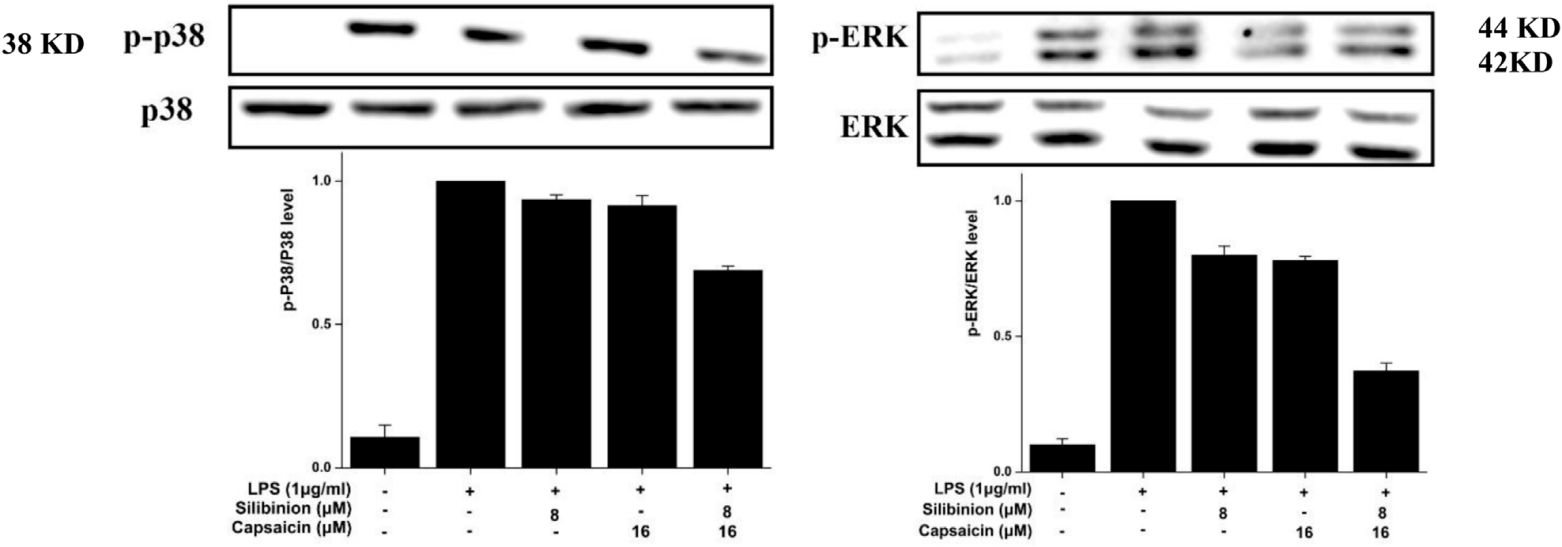
Effect of silibinin and capsaicin on the LPS-induced MAPK pathway. After separated by SDSPAGE (8%), transfer to the nitrocellulose membrane, and block for 1 h in non-fat milk (5%), the sample was incubated with the primary antibodies p-p38, p38, p-ERK, and ERK overnight at 4 °C, respectively, and subsequently incubation with the secondary antibody for 1 h.

Finally, the effect of silibinin and capsaicin on the LPS-induced MAPK pathway was assayed by evaluate the change of p-p38 and p-ERK. As can be seen in Figure 7, LPS treatment caused obvious over expressions on p-p38 that could be reduced by the treatment of silibinin (7.8% reduction) and capsaicin (9.4% reduction) alone. The Silibinin and capsaicin combination could effectively down-regulate the p-p38 expression (31.3% reduction). In contrast, silibinin, capsaicin, and silibinin in combination with capsaicin reduced p-ERK expression by 3.3, 4.9, and 62.3%, respectively. Previous reports indicated that silibinin and capsaicin could inhibit inflammation by suppressing the MAPK pathway activation. Our results also revealed that silibinin and capsaicin had a synergistic effect on the suppression of the LPS-induced MAPK pathway.

## Conclusion

**FIGURE 8:**
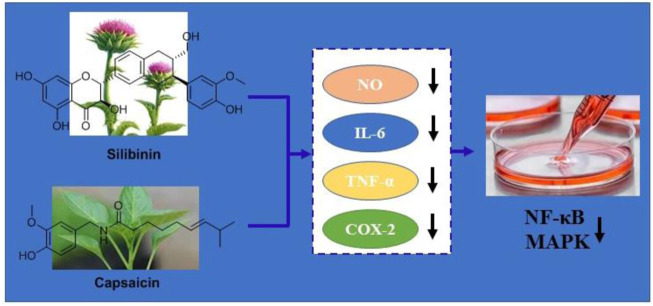
Schematic diagram of the anti-inflammation of the silibinin and capsaicin in combination.

In summary, we investigated the enhanced anti-inflammatory effects of the silibinin and capsaicin combination in LPS-induced RAW264.7 cells and the results showed that the silibinin and capsaicin combination had the best synergistic effect at a concentration ratio of 8–16 μM. The combination could effectively inhibit LPS- induced over the production of NO, TNF-α, IL-6, and COX-2. Their effective anti-inflammatory effect was related with the inhibiton of NF-κB and MAPK activities (Figure 8). Thus, it was concluded that the silibinin and capsaicin combination had a good anti-inflammatory effect and was expected to be used in the prevention and treatment of inflammation-related diseases.

## Data Availability

The original contributions presented in the study are included in the article/Supplementary Material, further inquiries can be directed to the corresponding author.
