# Supplementary material for: Enhanced Anti-Inflammatory Effects of Silibinin and Capsaicin Combination in Lipopolysaccharide-Induced RAW264.7 Cells by Inhibiting NF-κB and MAPK Activation
Source: Front Chem. 2022 Jun 30;10:934541. doi: 10.3389/fchem.2022.934541 (PMC9279934; doi:10.3389/fchem.2022.934541)
Supplement: Supplementary file 1 [file DataSheet1.docx]

**Supporting Information**

**WB** **Original Image**

**COX-2**

**

**

**β-actin**

**

**

**P65**

**

**

**laminb**





**p-p38**





**P38**





**p-ERK**





**ERK**

**

**
